# Metabolic syndrome and risk of ovarian cancer: a systematic review and meta-analysis

**DOI:** 10.3389/fendo.2023.1219827

**Published:** 2023-08-24

**Authors:** Ziyu Chen, Zesi Liu, Hongxia Yang, Chaosheng Liu, Fandou Kong

**Affiliations:** ^1^ Department of Obstetrics and Gynecology, The First Affiliated Hospital of Dalian Medical University, Dalian, Liaoning, China; ^2^ Department of Cardiology, The First Affiliated Hospital of Dalian Medical University, Dalian, Liaoning, China

**Keywords:** case-control studies, cohort studies, meta-analysis, ovarian cancer, metabolic syndrome

## Abstract

**Background:**

MetS is associated with greater morbidity and mortality in relation to a number of malignancies, but its association with ovarian cancer remains contested. The present study was a systematic review and meta-analysis of case-control and cohort studies examining the association between MetS and ovarian cancer risk.

**Methods:**

The study was registered on the PROSPERO platform in January 2023 (CRD42023391830). Up until February 13, 2023, a complete search was undertaken in PubMed, EMBASE, Web of Science, the Cochrane Library, and ClinicalTrials. On the basis of inclusion and exclusion criteria, eligible studies for meta-analysis were screened to determine the association between MetS and ovarian cancer risk.

**Results:**

Five studies were included in total, including three cohort studies and two case-control studies. Meta-analysis showed no significant correlation between metabolic syndrome and ovarian cancer (OR=1.29, 95% CI: 0.90-1.84). Significant heterogeneity (I^2^ = 92.6, P<0.05) existed between the included studies. We performed a subgroup analysis of the risk of bias and showed that only unadjusted stratification of risk of bias for smoking (OR= 3.19, 95% CI: 2.14-4.76) and hysterectomy (OR= 3.19, 95% CI: 2.14-4.76) demonstrated a relationship between metabolic syndrome and ovarian cancer risk. The meta-regression analysis revealed that smoking and hysterectomy excision were substantially linked with heterogeneity (p < 0.05).

**Conclusion:**

Our research revealed no statistically significant association between MetS and ovarian cancer risk. The prevalence of metabolic syndrome has highlighted the need of enhancing and controlling women’s metabolic health. However, the evaluation of metabolic syndrome as a cancer risk factor may be deceptive and etiologically uninformative.

## 1 Introduction

A combination of interconnected conditions known as the metabolic syndrome (MetS) is thought to significantly raise the risk of type 2 diabetes (T2DM) and cardiovascular disease. Its main components include hypertriglyceridemia, reduced HDL cholesterol concentration, hypertension, insulin resistance, and central obesity (1). Currently, ATP III and IDF are the two most prevalent definitions, but regardless of the criterion employed, prevalence estimates for the condition are practically identical in any given community (2). The frequency of metabolic syndrome (MetS) increases with age and is more likely to affect women than males, according to epidemiological research (3, 4). MetS relates to higher morbidity and mortality associated with a variety of malignancies, according to a rising number of studies. There is now theoretical evidence that the combined effects of MetS components like chronic inflammation and oxidative stress, as well as the bad effects of metabolism, increase the risk of cancer more than each MetS component alone (5).

With 22,000 cases reported each year in the United States, ovarian cancer is one of the most prevalent diseases in women and the fifth largest cause of cancer-related deaths in women (6, 7). There are numerous histological subtypes of ovarian cancer, each with distinct molecular alterations, clinical behaviours, and treatment outcomes. 90% of ovarian malignancies are EOC, which is typically diagnosed at an advanced stage and has a poor prognosis. The remaining 10% of ovarian cancer cases are non-epithelial ovarian cancer (NEOC), which consists primarily of germ cell tumours (GCT), sex cord mesenchymal tumours (SCST), and a few exceedingly rare tumours (8, 9). As far as we are aware, no meta-analyses have been performed to determine whether MetS raises the risk of ovarian cancer. At present, BRCA1 and BRCA2 germline mutations are the most significant genetic risk factors for EOC, with 6%-15% of women with EOC carrying these mutations. Furthermore, carriers of BRCA1 and BRCA2 are more sensitive to platinum-based chemotherapy than non-carriers and can be used to provide prognostic survival counselling. Despite the fact that the disease is typically diagnosed at more advanced stages and grades, the survival rate is higher (10). There is now a growing interest in modifiable risk factors for adverse outcomes, such as obesity, type 2 diabetes mellitus (T2DM), and other metabolic abnormalities. A growing number of studies have linked these conditions to an increased risk of ovarian cancer (11, 12).

Potentially significant ovarian cancer risk factors include metabolic disorders. Numerous research has investigated the association between specific MetS components and ovarian cancer, including obesity, dyslipidemia, and diabetes (13–15). However, comparatively few research has examined the relationship between MetS and ovarian cancer incidence. Various studies have conflicting information about the association between metabolic syndrome and ovarian cancer risk. Statistically significant correlations between metabolic syndrome and the risk of ovarian cancer have been established by certain research (16, 17), while others found no statistically significant correlation (18–20). Examination of their related research areas revealed no previously reported meta-analysis of MetS about ovarian cancer risk.

As a result, we conducted a methodical review and meta-analysis of case-control and cohort studies to examine the relationship between MetS and ovarian cancer risk.

## 2 Materials and methods

### 2.1 Study design and search strategy

The systematic evaluation and meta-analysis of the study were conducted in accordance with the PRISMA statement’s requirements (21). At the same time, the study was registered on the PROSPERO platform in January 2023 (CRD42023391830).

The most recent recommendations were used to conduct the literature search (22). The search was conducted in order to locate as many articles as possible that discussed the link between MetS and the risk of ovarian cancer. The **Supplementary Material** includes search measurements. Through a thorough search in PubMed, EMBASE, Web of Science, the Cochrane Library, and ClinicalTrials, we were able to find every publication that might be pertinent through February 13, 2023.

### 2.2 Selection criteria

Included in our meta-analysis were only the publications that matched the following criteria (1): cohort studies and case-control studies (2); examined the connection between MetS and ovarian cancer risk (3); provided risk estimates (relative risks, odds ratios, hazard ratios) and 95% confidence intervals. Articles that fit any of the following requirements were disqualified (1): reviews, conference abstracts, books, reports, and commentaries (2); studies from which exact data extraction was not possible (3); for studies that have been published more than once, data from the information with the most thorough reporting and longest follow-up. Two researchers independently reviewed titles and abstracts to exclude ineligible publications. All disagreements were settled through conversation and cooperation with a third reviewer. The examination of the full text was performed independently by the same reviewers, and all discrepancies were resolved by discussion and contact with a third reviewer.

### 2.3 Data extraction and quality assessment

Two researchers independently collected pertinent information from the included study data using a standard form that was created prior to data extraction and modified for the data extracted twice. The following data were taken out: first author, year of publication, country of publishing, study design, study period, study source, adjustment factors, mean age (or range), amount of patients, number of ovarian cancer cases, diagnostic standards for MetS, risk estimates, and 95% CIs.

Using the Newcastle-Ottawa scale (NOS), two researchers independently assessed the caliber of the studies that were included (23). The NOS contains 8 items consisting of three domains: selection of study population (0 to 4), comparability between groups (0 to 2), and outcome measures (0–3). High-quality studies had a total score ≥7.

### 2.4 Statistical analysis

Considering the small number of included studies and the increasing “rank” of ORs, RRs, and HRs, the RRs and HRs were directly treated as ORs (24, 25). To analyze the link between MetS and ovarian cancer risk, we utilized a random effects model to obtain pooled risk estimates for each study included. We evaluated heterogeneity with the Cochran Q test and the I^2^ statistic. Subgroup and meta-regression analyses were conducted to identify the causes of study heterogeneity and the significance of OR variations between subgroups. Sensitivity analysis investigated if excluding a study would significantly change the total OR. We did not employ statistical techniques to test for publication bias since less than ten studies were included (26). p values < 0.05 indicate statistical significance. All analyses were conducted using Stata version 17 (StataCorp., College Station, TX, USA).

## 3 Results

### 3.1 Literature search

**Figure 1:**
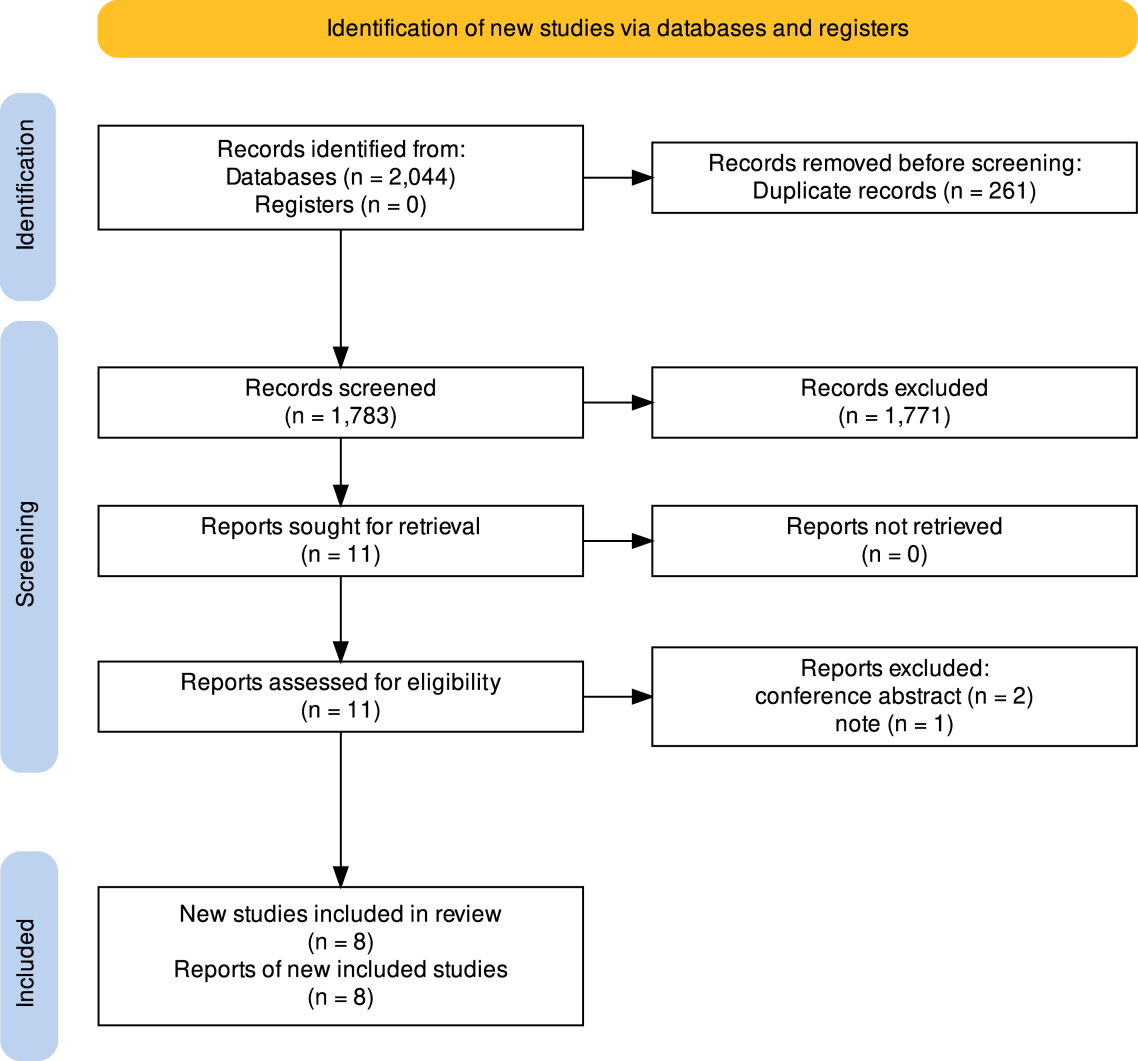
Flowchart of included studies for the meta-analysis.

As illustrated in **Figure 1**, a complete search of PubMed, EMBASE, Web of Science, the Cochrane Library, and ClinicalTrials yielded 2,044 results. By considering the inclusion and exclusion criteria, as well as removing duplicates, we excluded 2,036 records. Then, eight records were carefully reviewed through the full text, and five studies were finally discovered and included in our meta-analysis. Two of the researches reported epithelial ovarian cancer, another study referred to ovarian and fallopian tube cancer collectively as “ovarian cancer,” and two studies reported patients with various benign and malignant tumors.

### 3.2 Study characteristics and quality assessment

**Table 1** provides a summary of the characteristics of the five included studies. These research, published between 2011 and 2020, included three cohort studies (18–20) and two case-control studies (16, 17). 5 studies were conducted in different countries, namely the UK, China, Norway, Korea, and the USA.

**Table 1:** Characteristics of studies on the presence of metabolic syndrome and ovarian cancer risk.

Author	Year	Country	Study design	Study period	Mean age(or range)[year]	Cases/Sample size	Diagnostic criteria for MetS
Cao	2020	UK	cohort	2006-2016	56.3	NA/206954	ATP-III
Chen	2017	China	case-control	2010-2016	Intervene:52.59±9.20 Control:52.97±9.73	Intervene:144/573 Control:79/1146	CDS
Bjørge	2011	Norway	cohort	1974-2005	44	644/287320	NA
Ko	2016	Korea	cohort	2002-2013	20s-60s	82/37807	(1) obesity, defined as a body mass index ≥25 kg/m2; (2) dysfunction of glucose regulation, defined as a fasting glucose level ≥100 mg/dL or a history of type 2 diabetes; (3) dyslipidemia, defined as a serum total cholesterol level ≥200 mg/dL; (4) hypertension, defined as blood pressure ≥130/85 mmHg or a history of hypertension.
Michels	2019	US	case-control	1994-2013	68-89	Intervene:3751/16850 Control:65041/281878	ATP-III

MetS, metabolic syndrome; ATP-III, Adult Treatment Panel III of the National Cholesterol Education Program; CDS, Chinese Diabetes Society.

A summary of the methodological quality assessment of the included studies is shown in **Supplementary Tables 1, 2**. All studies were of high quality according to the NOS, with two studies receiving a score of 7 (16, 19), two studies receiving a score of 8 (17, 18), and even one study receiving a score of 9 (20).

### 3.3 MetS and ovarian cancer risk

**Figure 2:**
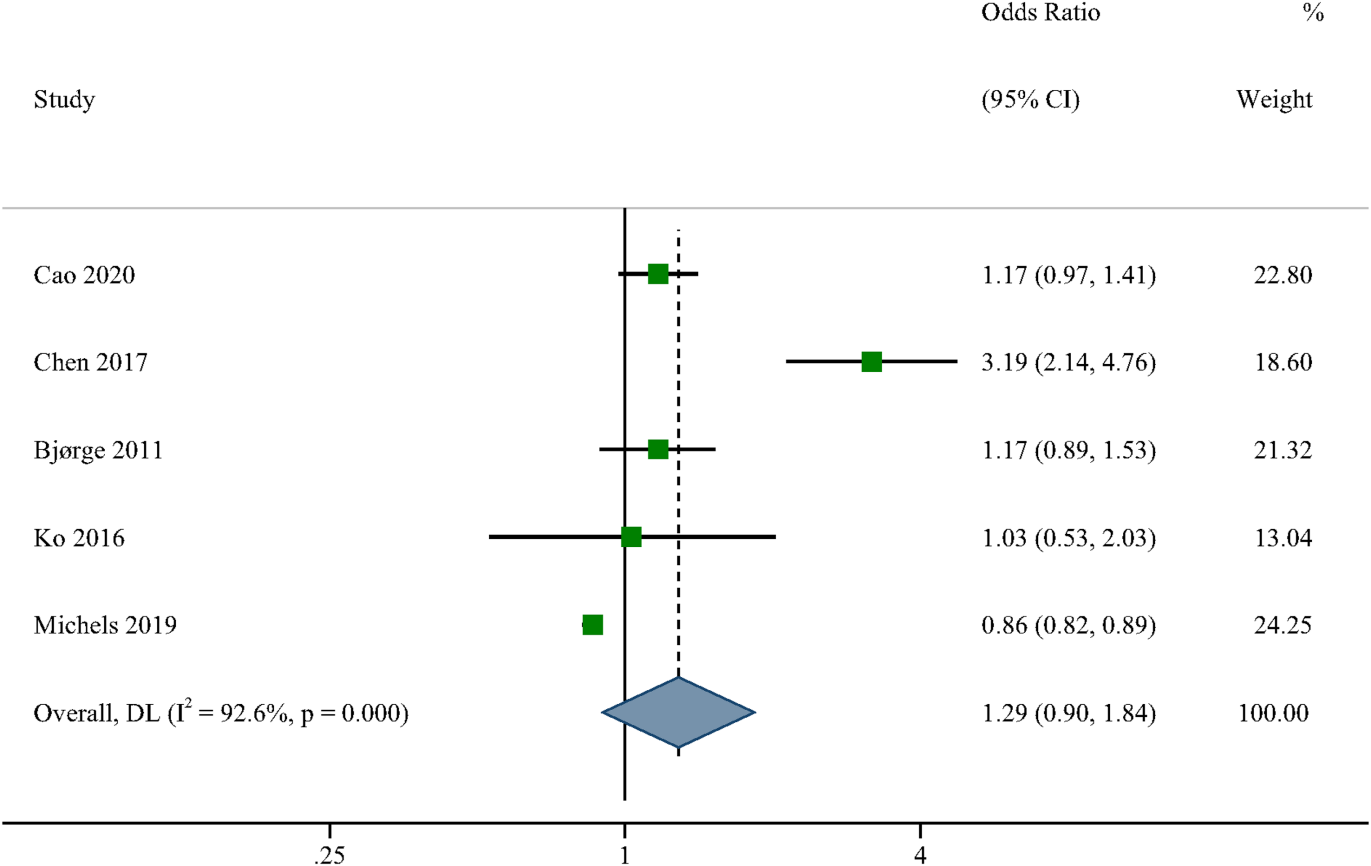
Forest plots (random effect model) of meta-analysis on the association between the presence of metabolic syndrome and ovarian cancer risk. Squares indicate study-specific ORs (size of the square reflects the study-specific statistical weight); horizontal lines indicate 95% CIs; diamond indicates the summary OR with its 95% CI. OR, odds ratio; CI, confidence interval.

A random-effects meta-analysis of the five studies indicating a connection between MetS and ovarian cancer risk (**Figure 2**) revealed no statistically significant correlation between MetS and ovarian cancer (OR=1.29, 95% CI: 0.90-1.08). There was substantial heterogeneity amongst the included studies (I^2 =^ 92.6, P0.05). As a consequence, we performed a study-by-study exclusion method to remove individual studies one by one, with no significant change in risk estimates (**Supplementary Figure 1**).

### 3.4 Subgroup analyses and meta-regression

**Figure 3:**
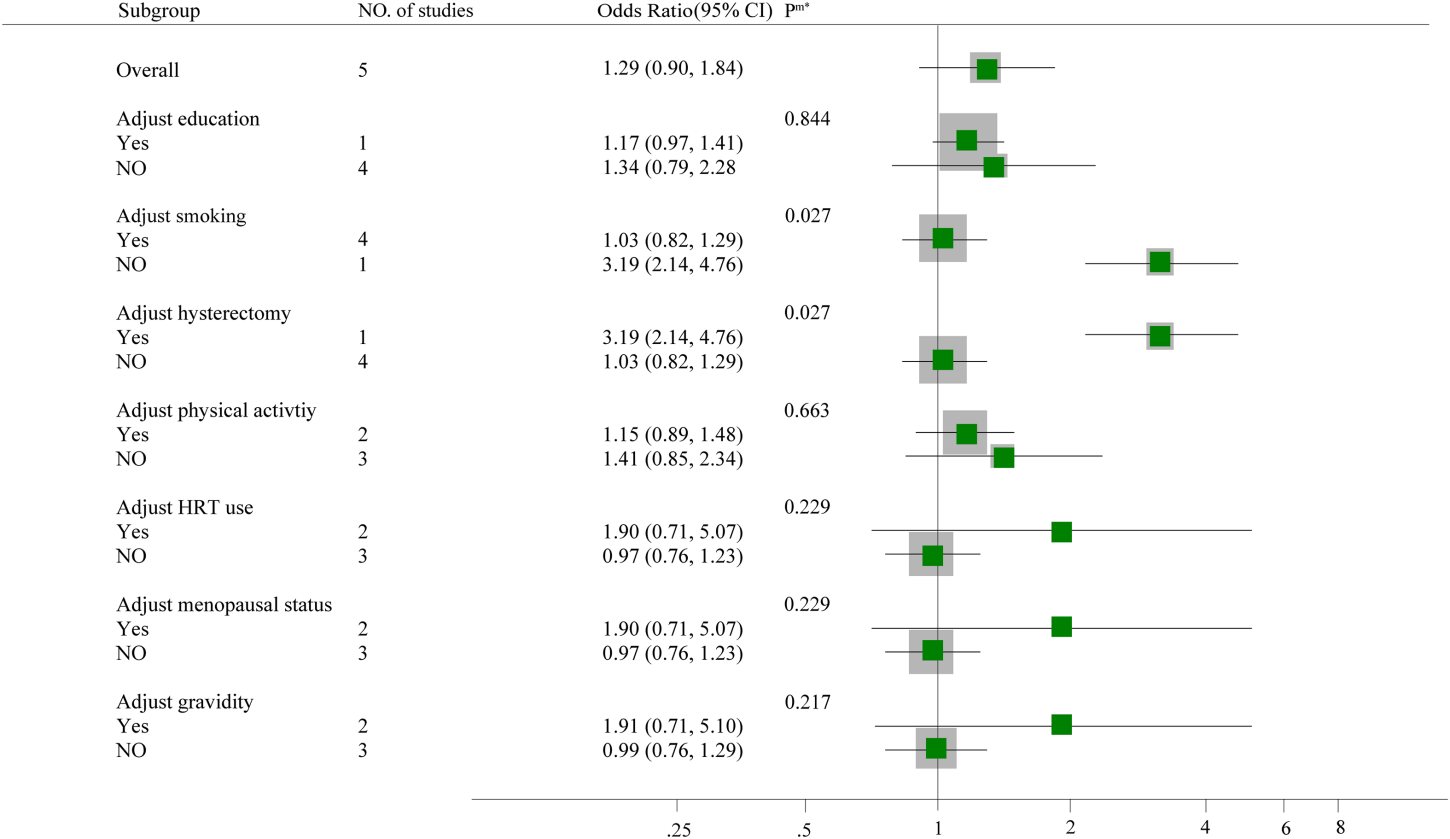
Subgroup analyses and meta-regression for the association between the presence of metabolic syndrome and ovarian cancer risk.

We performed a subgroup analysis of the risk of bias and showed that only the stratum of unadjusted risk of bias for smoking (OR= 3.19, 95% CI: 2.14-4.76) and hysterectomy (OR= 3.19, 95% CI: 2.14-4.76) showed an association between metabolic syndrome and ovarian cancer risk, and its number of studies was limited. However, most of the remaining strata showed no correlation between the two. meta-regression showed a significant correlation between smoking, hysterectomy and heterogeneity among the selected study characteristics (p < 0.05). The correlation results are shown in **Figure 3**.

## 4 Discussion

Several latest studies have studied the relationship between MetS and the ovarian cancer risk with contradictory results. For the random-effects meta-analysis, we looked through PubMed, EMBASE, Web of Science, the Cochrane Library, and ClinicalTrials to find 5 studies that indicated the relationship. Our investigation revealed no correlation between metabolic syndrome and the risk of ovarian cancer. This result was durable in sensitivity analyses and was mostly consistent among subgroups defined by various research parameters. As far as we are aware, no meta-analyses have been performed to determine whether MetS raises the risk of ovarian cancer.

According to reports, metabolic syndrome is a significant component in the advancement of multiple forms of cancer (27, 28). Fewer research have examined the association between ovarian cancer risk and metabolic syndrome than for other malignancies. Nevertheless, several research have investigated the relationship between MetS and its components and ovarian cancer. There have been studies indicating a link between obesity and the progression of ovarian cancer. The Ovarian Cancer Epidemiology Study Collaborative Group conducted a meta-analysis of 47 studies that included 25,157 ovarian cancer cases and indicated a 5% increase in the incidence of ovarian cancer per 10 kg/m^2^ (13). A retrospective cohort study of 70,258 Chinese women identified a 2-fold increased risk of ovarian cancer among women with a BMI of 30 or higher (29). According to a meta-analysis of 14 studies conducted by Protan et al. (12), the survival rate of obese women was marginally lower than that of non-obese women (HR=1.17, 95% CI: 1.03- 1.34). The prognosis following cancer treatment may be impacted by aspects of MetS outside the metabolic component. Diabetes was related with increased recurrence and mortality in women with ovarian cancer, according to an analysis of 367 individuals with epithelial ovarian cancer in the United States by Shah et al. (14). Xiao Hu et al. discovered that lower CA125 concentrations in study subjects with higher BMI. Actually, obesity was associated with lower CA125 levels, possibly due to plasma volume’s diluting effect (30). The researchers of the Me-Can trial noticed that baseline hypertension enhanced the likelihood of endometrioid cancers (31). Zhang et al. demonstrated a link between high blood cholesterol and an increased risk of ovarian cancer (15).

Meta-analyses have been performed showing that study results vary considerably by study design, population age, tissue type, and timing and type of exposure assessment (e.g., BMI versus waist circumference). Obesity and type 2 diabetes may raise the probability of ovarian cancer, however the relationships were weak, even minor, and heterogeneous (32, 33). These findings revealed that specific metabolic components are associated with ovarian cancer, and that the correlations between these components may differ in direction and may be influenced by the prevalence of these components in the research group. As a consequence, it is biologically and statistically inappropriate to speak of these combinations as a composite variable in ovarian cancer studies.

This analysis has the advantage of examining the association between MetS and ovarian cancer risk by the inclusion of 5 observational studies that were carefully screened. Additionally, our findings were consolidated by sensitivity analysis. Third, our study was significantly heterogeneous. Therefore, comprehensive subgroup and univariate meta-regression analyses were conducted to determine if the results differed based on important research features. Finally, we assessed each piece thoroughly to ensure that they were of adequate quality to be included.

Notably, the study is limited in several areas. First, all meta-analyses, especially those of observational studies, have historically been concerned with clinical and methodological heterogeneity (34). Despite the use of meta-regression analysis, the small sample size may have hindered its ability to identify causes of heterogeneity. Second, even though the included studies tried to control for known risk factors and we got the most accurate risk estimates we could, residual confounding can’t be ruled out because our results came from observational studies, which always had residual confounding (34). Thirdly, due to the introduction of too little inclusion literature, the study directly combined RR and HR as OR. Finally, because we had <10 included studies, we did not use funnel plots or Egger regression tests to evaluate publication bias.

## 5 Conclusions

Our research revealed no statistically significant association between MetS and ovarian cancer risk. The prevalence of metabolic syndrome has highlighted the need of enhancing and controlling women’s metabolic health. However, the evaluation of metabolic syndrome as a cancer risk factor may be deceptive and etiologically uninformative.

## Data availability statement

The original contributions presented in the study are included in the article/**Supplementary Material**. Further inquiries can be directed to the corresponding authors.

## Author contributions

All authors are solely responsible for the content and writing of the manuscript. All authors made significant contributions to the design, data collection and interpretation, and manuscript preparation and revision of this study. All authors contributed to the article and approved the submitted version.

## Supplementary material

Supplementary Figure 1 Sensitivity analysis of studies investigating the association between the presence of metabolic syndrome
