# Supplementary figures and images for: Metabolic syndrome and risk of ovarian cancer: a systematic review and meta-analysis
Source: Front Endocrinol (Lausanne). 2023 Aug 24;14:1219827. doi: 10.3389/fendo.2023.1219827 (PMC10484223; doi:10.3389/fendo.2023.1219827)

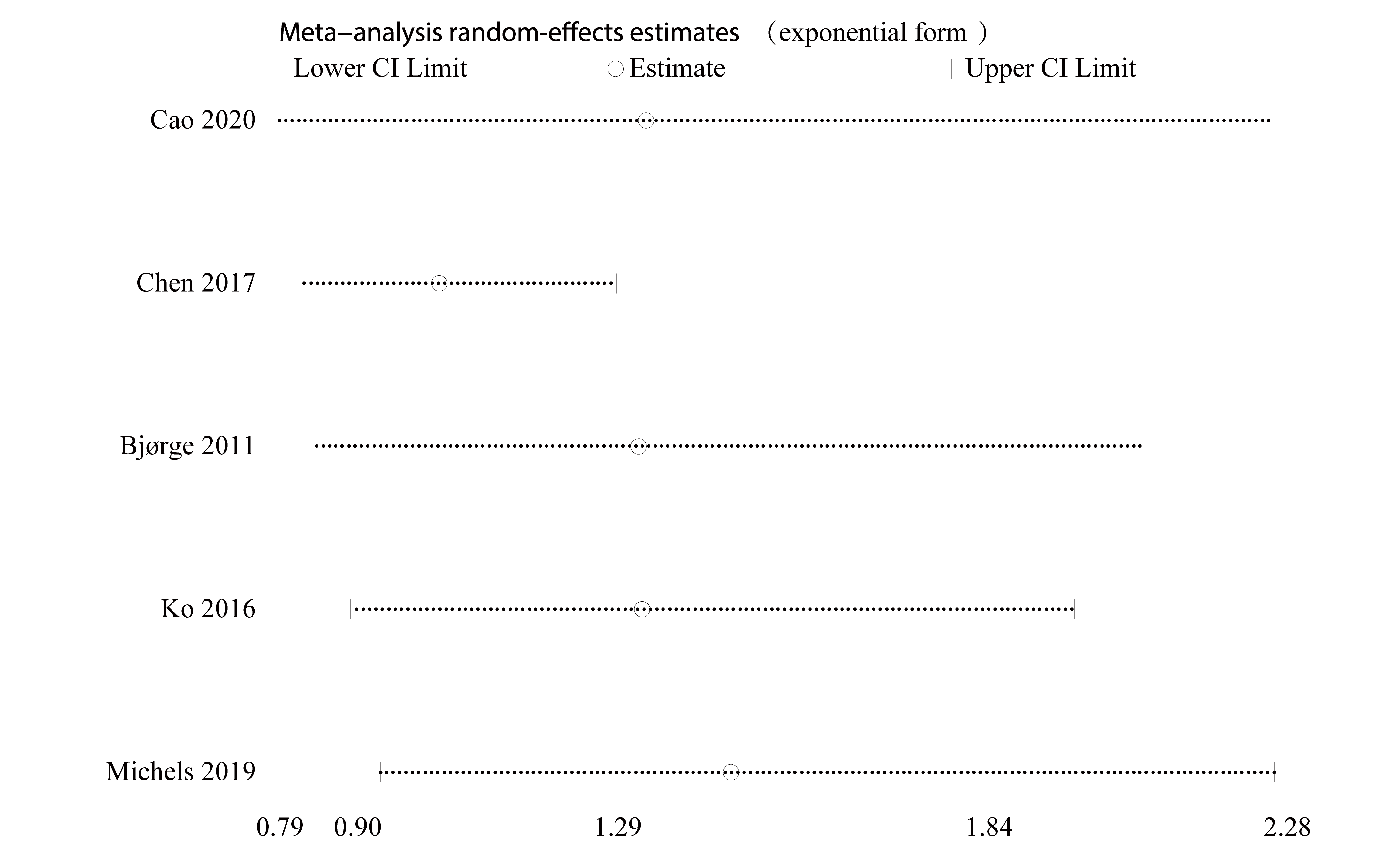

Supplement: Supplementary Figure 1 — Sensitivity analysis of studies investigating the association between the presence of metabolic syndrome [file Image_1.tif]
