# Supplementary material for: Metabolic syndrome and risk of ovarian cancer: a systematic review and meta-analysis
Source: Front Endocrinol (Lausanne). 2023 Aug 24;14:1219827. doi: 10.3389/fendo.2023.1219827 (PMC10484223; doi:10.3389/fendo.2023.1219827)
Supplement: Supplementary file 2 [file DataSheet_1.docx]

Search measurements：

pubmed：

("Metabolic Syndrome"[Title/Abstract] OR "syndrome x"[Title/Abstract] OR "MetS"[Title/Abstract] OR "insulin resistance syndrome"[Title/Abstract] OR "Metabolic X Syndrome"[Title/Abstract] OR "Metabolic Cardiovascular Syndrome"[Title/Abstract] OR "cardio metabolic syndrome"[Title/Abstract] OR "Metabolic Syndrome"[MeSH Terms]) AND ("Ovarian Neoplasms"[MeSH Terms] OR ("ovar*"[Title/Abstract] AND ("cancer*"[Title/Abstract] OR "neoplas*"[Title/Abstract] OR "tumor*"[Title/Abstract] OR "tumour*"[Title/Abstract] OR "carcinoma*"[Title/Abstract] OR "adenocarcinoma*"[Title/Abstract] OR "malignan*"[Title/Abstract])) OR ("Cancer of Ovary"[Title/Abstract] OR "Cancer of the Ovary"[Title/Abstract]))

cochrane library：

#1 MeSH descriptor: [Ovarian Neoplasms] explode all trees

#2 (ovar* near/5 (cancer* OR neoplas* OR tumor* OR tumour* OR carcinoma* OR adenocarcinoma* OR malignan*)):ti,ab,kw

#3 #1 OR #2

#4 MeSH descriptor: [Metabolic Syndrome] explode all trees

#5 (Metabolic syndrome):ti,ab,kw OR (syndrome x):ti,ab,kw OR (MetS):ti,ab,kw OR (insulin resistance syndrome):ti,ab,kw

#6 ("Metabolic X Syndrome"):ti,ab,kw OR ("Metabolic Cardiovascular Syndrome"):ti,ab,kw OR (cardio-metabolic syndrome):ti,ab,kw

#7 #4 OR #5 OR #6

#8 #3 AND #7

EMBASE：

#1 'metabolic syndrome x'/exp

#2 'metabolic syndrome':ab,ti OR 'syndrome x':ab,ti OR mets:ab,ti OR 'insulin resistance syndrome':ab,ti OR 'metabolic x syndrome':ab,ti OR 'metabolic cardiovascular syndrome':ab,ti OR 'cardio-metabolic syndrome':ab,ti

#3 #1 OR #2

#4 'ovary tumor'/exp

#5 'ovar* adj5':ab,ti AND (cancer*:ab,ti OR neoplas*:ab,ti OR tumor*:ab,ti OR tumour*:ab,ti OR carcinoma*:ab,ti OR adenocarcinoma*:ab,ti OR malignan*:ab,ti) AND .mp.:ab,ti

#6 #4 OR #5

#7 #3 AND #6

Web of science：

#1 TS=(Ovarian Neoplasms) OR TS=(ovar* AND (cancer* OR neoplas* OR tumor* OR tumour* OR carcinoma* OR adenocarcinoma* OR malignan*)) OR TS=("Cancer of Ovary") OR TS=("Cancer of the Ovary")

#2 TS=(Metabolic Syndrome) OR TS=(syndrome x) OR TS=(MetS) OR TS=(insulin resistance syndrome) OR TS=(Metabolic X Syndrome) OR TS=(Metabolic Cardiovascular Syndrome) OR TS=(cardio metabolic syndrome)

#3 #1 AND #2
