# Supplementary material for: Metabolic syndrome and risk of ovarian cancer: a systematic review and meta-analysis
Source: Front Endocrinol (Lausanne). 2023 Aug 24;14:1219827. doi: 10.3389/fendo.2023.1219827 (PMC10484223; doi:10.3389/fendo.2023.1219827)
Supplement: Supplementary file 3 [file Table_1.docx]

| Study | Selection | | | | Comparability | | Exposure | | | | Scores |
| --- | --- | --- | --- | --- | --- | --- | --- | --- | --- | --- | --- |
|  | Adequate definition of cases | Representativeness of the cases | Selection of Controls | Definition of Controls | | Comparability of cases and controls on the basis of the design or analysis | | Ascertainment of exposure | Same method of ascertainment for cases and controls | Non-Response rate |  |
| Chen 2017 | ☆ | ☆ | ☆ | ☆ | | ☆☆ | |  | ☆ |  | 7 |
| Michels 2019 | ☆ | ☆ | ☆ | ☆ | | ☆☆ | | ☆ | ☆ |  | 8 |

Supplementary Table 1. Results of the critical evaluation of included studies using the Newcastle–Ottawa Quality Assessment Scale for case-control studies.
