# Supplementary material for: Metabolic syndrome and risk of ovarian cancer: a systematic review and meta-analysis
Source: Front Endocrinol (Lausanne). 2023 Aug 24;14:1219827. doi: 10.3389/fendo.2023.1219827 (PMC10484223; doi:10.3389/fendo.2023.1219827)
Supplement: Supplementary file 4 [file Table_2.docx]

Supplementary Table 2. Results of the critical evaluation of included studies using the Newcastle–Ottawa Quality Assessment Scale for cohort studies

| Study | Selection | | | | | Comparability | | Outcome | | | Scores |
| --- | --- | --- | --- | --- | --- | --- | --- | --- | --- | --- | --- |
|  | Representativeness of the exposed cohort | Selection of the non exposed cohort | Ascertainment of exposure | Demonstration that outcome of interest was not present at start of study | Comparability of cases and controls on the basis of the design or analysis | | Assessment of outcome | | Was follow-up long enough for outcomes to occur | Adequacy of follow up of cohorts |  |
| Cao 2020 | ☆ | ☆ | ☆ | ☆ | ☆☆ | | ☆ | | ☆ |  | 8 |
| Bjørge 2011 | ☆ | ☆ | ☆ | ☆ | ☆ | | ☆ | | ☆ |  | 7 |
| Ko 2016 | ☆ | ☆ | ☆ | ☆ | ☆☆ | | ☆ | | ☆ | ☆ | 9 |
